# Transcatheter Aortic Valve Replacement is Associated With a Lower Risk of Aortic Dissection Than Surgical Aortic Valve Replacement: A Propensity Score-Matched Analysis

**DOI:** 10.31083/RCM50574

**Published:** 2026-07-23

**Authors:** Mohamed Doma, Mangesh Kritya, Adham Ramadan, Ibrahim Kamel, Iuri Ferreira Felix, Seif Shlkamy, Shanmukh Sai Pavan Lingamsetty, Ahmed K. Mahmoud, Mahmoud Ismayl, Ashwin Mahesh, Andrew M. Goldsweig

**Affiliations:** ^1^Cardiovascular Research Center, Massachusetts General Hospital, Harvard Medical School, Boston, MA 02114, USA; ^2^DeBakey Heart and Vascular Institute, Houston Methodist, Houston, TX 77030, USA; ^3^Department of Internal Medicine, Boston Medical Center, Boston, MA 02118, USA; ^4^Department of Medicine, Mayo Clinic, Rochester, MN 55905, USA; ^5^Department of Medicine, Alexandria Faculty of Medicine, 5372066 Alexandria, Egypt; ^6^Division of Gastroenterology and Hepatology, Beth Israel Deaconess Medical Center, Harvard Medical School, Boston, MA 02215, USA; ^7^Department of Cardiovascular Medicine, Mayo Clinic, Rochester, MN 55905, USA; ^8^Department of Cardiology, University of Pittsburgh Medical Center, Harrisburg, PA 15213, USA; ^9^Department of Cardiovascular Medicine, Baystate Medical Center and Division of Cardiovascular Medicine, University of Massachusetts-Baystate, Springfield, MA 01199, USA

**Keywords:** aortic dissection, transcatheter aortic valve replacement, surgical aortic valve replacement, aortic valve disease

## Abstract

**Background:**

Aortic valve replacement is the primary treatment for aortic valve disease and can be performed using either transcatheter (TAVR) or surgical (SAVR) approaches. The risk of aortic dissection (AD) with TAVR compared with SAVR remains unclear. We aimed to compare AD risk between TAVR and SAVR using a large, real-world cohort.

**Methods:**

We conducted a retrospective cohort analysis using the TriNetX research network. Patients with a diagnosis of aortic stenosis, aortic insufficiency, mixed aortic valve disease (AVD), or unspecified AVDwho underwent either TAVR or SAVR were included. A 1:1 propensity score-matching (PSM) based on baseline characteristics was performed to adjust for potential confounding. Logistic regression and Cox proportional hazards models were used to estimate odds ratios (ORs) and hazard ratios (HRs), with 95% confidence intervals (CIs).

**Results:**

Among 95,043 patients undergoing isolated aortic valve replacement, 20,237 matched pairs were analyzed after 1:1 PSM. Baseline characteristics were well balanced, with a mean age of 73.3 ± 9.3 years in the TAVR group and 73.2 ± 8.5 years in the SAVR group; 60.8% and 60.7% were male, respectively. TAVR was associated with significantly lower odds of composite AD than SAVR at all time points (1-year OR 0.60; 95% CI 0.44–0.82; 3-year OR 0.64; 95% CI 0.48–0.83; 5-year OR 0.66; 95% CI 0.51–0.85; *p* < 0.01 for all). Stratified analyses demonstrated lower odds of both Stanford type A and type B AD at all time points. Kaplan–Meier analysis demonstrated a separation in composite AD-free survival between the two groups (HR 0.66; 95% CI 0.51–0.86; *p* = 0.002).

**Conclusion:**

In this large, real-world cohort, despite a low event rate, TAVR was associated with a significantly lower likelihood of composite AD than SAVR.

## 1. Introduction

Aortic dissection (AD) is a rare but life-threatening cardiovascular emergency characterized by a tear in the intimal layer of the aorta, leading to the creation of a false lumen [1]. This condition can rapidly progress to serious adverse outcomes, including aortic rupture, organ malperfusion, and death if not promptly diagnosed and managed [2]. Despite advances in imaging and management strategies, AD continues to carry significant morbidity and mortality rates, highlighting the importance of identifying and controlling procedural risk factors that may precipitate this event [3,4,5,6].

Transcatheter aortic valve replacement (TAVR) has emerged as a transformative therapy for patients with aortic valve disease (AVD) [7]. Initially reserved for patients deemed inoperable or at high surgical risk, TAVR has expanded to intermediate and low-risk populations due to technological refinements and growing evidence of its safety and efficacy [8,9]. Although procedural complications such as vascular injury and paravalvular leak have been extensively studied in the context of TAVR, the incidence and risk of AD following TAVR remain less well characterized [10].

Surgical aortic valve replacement (SAVR), the traditional standard of care for AVD, involves open-heart surgery and direct replacement of the diseased valve [11]. While SAVR provides durable hemodynamic outcomes, it carries risks inherent to open surgical procedures, including those related to aortic manipulation, such as AD [12].

Although a rare vascular complication of aortic valve replacement (AVR) [10], AD is often fatal. Direct comparisons of the incidence of AD between TAVR and SAVR remain limited in the literature. SAVR, requiring not only aortic manipulation but actual aortic incision and suturing, may be associated with AD not only during the perioperative period, but also during long-term follow-up. Even though TAVR adoption evolved substantially over the study period, with earlier use in higher-risk patients and later expansion to intermediate- and low-risk groups, the use of propensity score-matching (PSM) to construct cohorts balanced across demographics, comorbidities, and established AD risk factors enables meaningful comparison of long-term outcomes across the full spectrum of TAVR and SAVR patients treated in contemporary practice.

This study aims to compare the mid- and long-term risk of AD between patients undergoing TAVR versus SAVR using a large, real-world dataset and robust statistical techniques to provide estimates of risk differences between the two procedures to guide care for future patients.

## 2. Methods

### 2.1 Study Design and Data Source

This retrospective cohort study was conducted using the TriNetX Research Network, a federated health research platform aggregating de-identified electronic health records from over 170 healthcare organizations internationally. The network provides longitudinal access to clinical data, including diagnoses, procedures, medications, and laboratory values. At the time of data extraction, 80 institutions contributed data. TriNetX collects and aggregates data in real-time. Each patient’s data is linked longitudinally within each participating institution through a unique, encrypted identifier. Data are not linked across different health systems; thus, events occurring outside the contributing institutions may not be captured. The data query for this study was executed on April 14, 2026, and included all data available in the registry from January 2012 to December 2022. This study adhered to the STROBE (Strengthening the Reporting of Observational Studies in Epidemiology) guidelines for cohort studies (**Supplementary Table 1**). TriNetX systematically excludes patients with missing key data fields, so missing data was not a limitation in this analysis. This study was conducted on a retrospective and de-identified database and determined to be exempt from ethics approval of informed consent. No patient-level identifiable information was accessed.

### 2.2 Cohort Identification and Definitions

Adult patients aged ≥18 years with a diagnosis of AS, aortic insufficiency, mixed AVD, or unspecified AVD who underwent either TAVR or SAVR were initially identified. Two cohorts were defined: patients who underwent isolated TAVR and those who underwent isolated SAVR. The index date was defined as the date of the valve replacement procedure. Baseline characteristics were extracted 1 day prior to the index event. The definitions of baseline characteristics are shown in **Supplementary Table 2**.

Patients were excluded if they had a prior diagnosis of AD, a history of connective tissue or vascular disorders, or a prior or concomitant aortic procedure. To ensure cohort exclusivity, patients with a history of TAVR were excluded from the SAVR cohort, and patients with a history of SAVR were excluded from the TAVR cohort. Full details of inclusion and exclusion criteria are presented in **Supplementary Table 3**. Patients were followed beginning one day after the index procedure and up to 5 years. Full details of follow-up at each time point are reported in **Supplementary Table 4**.

### 2.3 Outcomes

The primary study outcome was the composite incidence of any AD over 1, 3, and 5 years. Secondary outcomes included AD stratified by Stanford classification: Stanford Type A and Stanford Type B. All endpoints were identified using standardized diagnostic codes within participating institutions (**Supplementary Table 5**).

### 2.4 Statistical Analysis

PSM was performed using TriNetX’s built-in greedy nearest-neighbor algorithm with a caliper of 0.1, based on demographic characteristics, comorbid conditions, clinical variables, and preexisting risk factors for AD; full details of matching covariates are presented in **Supplementary Methods 1**. Covariate balance was assessed using standardized differences (Std. diff.), with values <0.1 considered acceptable; *p*-values > 0.05 were considered statistically non-significant. Logistic regression was performed to estimate odds ratios (ORs) with 95% confidence intervals (CIs) were calculated to compare the likelihood of all study outcomes (composite AD, Stanford Type A and Stanford Type B ADs) between the TAVR and SAVR groups at 1, 3, and 5 years. To account for differences in follow-up time and censoring, hazard ratios (HRs) were estimated for all study outcomes using a Cox proportional hazard model.

Time-to-event analyses were conducted using Kaplan–Meier curves and compared using the log-rank test; all-cause mortality was considered a competing event, and numbers of survivors at each annual timepoint were estimated as the total matched cohort minus cumulative all-cause mortality.

In addition to the PSM analysis, multivariable Cox proportional hazards regression was performed for all outcomes assessed in the unmatched cohort, adjusting for the same covariates used in the PSM model. This approach was undertaken to account for potential loss of sample size after matching and to evaluate the consistency of findings in the full study population. All analyses were conducted within the TriNetX platform. Figures were generated in R version 4.3.2 (R Foundation for Statistical Computing, Vienna, Austria) using the ggplot2 package.

## 3. Results

### 3.1 Characteristics of the Study Population

**Fig. 1.**
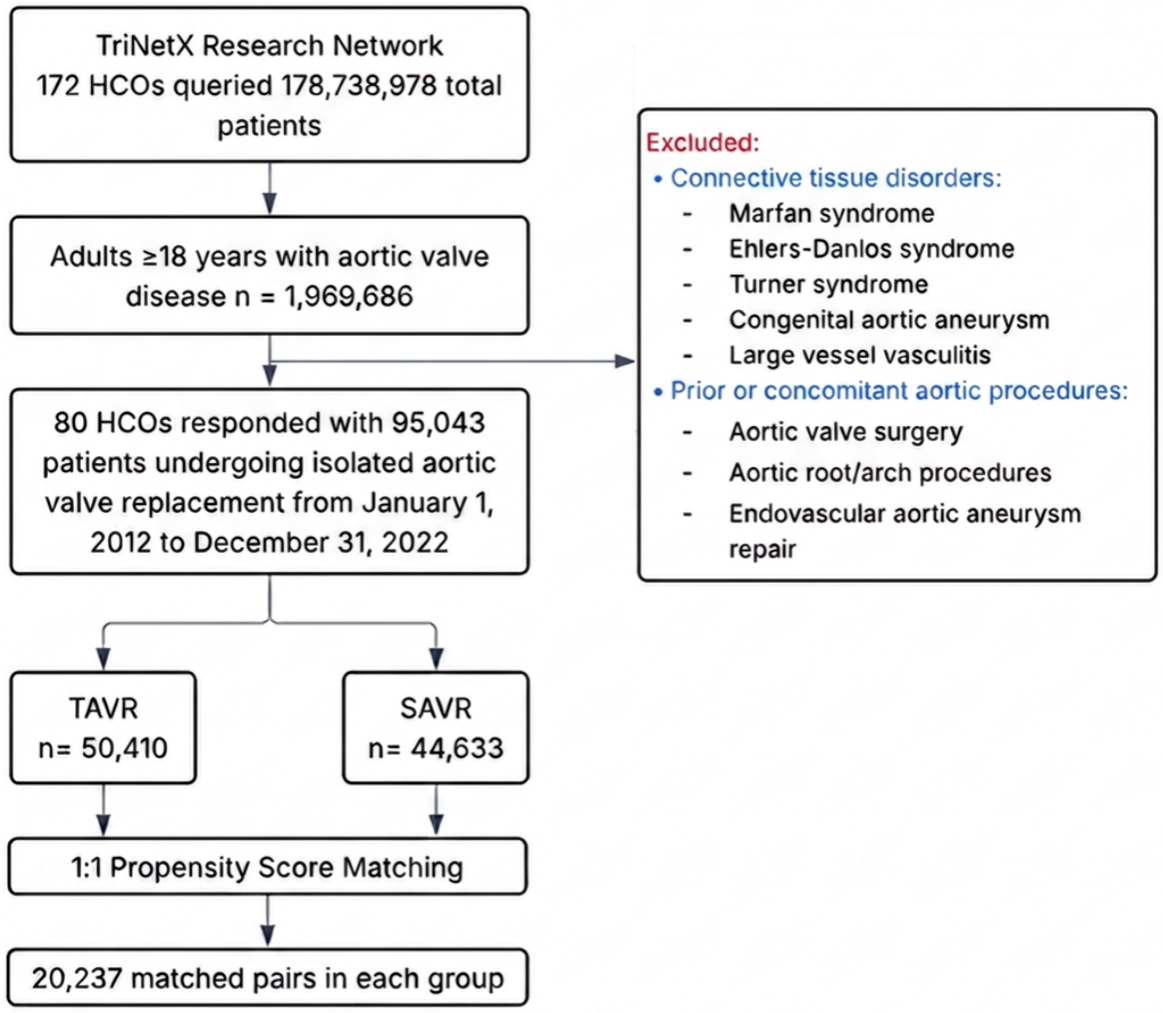
**Study population flowchart**. HCO, health care organization; SAVR, surgical aortic valve replacement; TAVR, transcatheter aortic valve replacement.

Among 95,043 patients who underwent isolated AVR from January 2012 to December 2022, 50,410 (53.0%) underwent TAVR and 44,633 (47.0%) underwent SAVR. The study design and patient flow are summarized in Fig. 1. Before matching, TAVR patients were older and had a higher prevalence of comorbidities, including heart failure, chronic kidney disease, atrial fibrillation, and peripheral vascular disease. In contrast, SAVR patients were more likely to be male and to have a bicuspid aortic valve, aortic aneurysms, and nicotine dependence.

After 1:1 PSM, the final cohort included 20,237 patients in each group. The mean age was 73.3 ± 9.3 years in the TAVR group and 73.2 ± 8.5 years in the SAVR group. Males comprised 60.8% of the TAVR group and 60.7% of the SAVR group. Racial distribution was similar across both groups, with approximately 80% identifying as White.

Baseline comorbidities and laboratory values were well balanced between the groups following matching, with standardized differences below 0.1 for all variables. Although low-density lipoprotein (LDL) cholesterol, body mass index (BMI), creatinine, activated partial thromboplastin time (aPTT), and prothrombin time (PT) showed statisticaly significant *p*-values after matching, the std. diff were small and remained well below 0.1, consistent with a large-sample statistical artifact rather than a clinically meaningful imbalance. To further address this, these variables were additionally analyzed as binary thresholds: LDL ≥160 mg/dL, BMI ≥30 kg/m^2^, creatinine ≥1.2 mg/dL, aPTT ≥35 seconds, and PT ≥13.5 seconds, none of which differed significantly between groups after matching. A comprehensive summary of all variables before and after matching is presented in Table 1.

**Table 1.** **Baseline characteristics and demographics before and after propensity score-matching**.

Variable		Before PSM				After PSM			
		TAVR (n = 50,410)	SAVR (n = 44,633)	*p*-value	Std. diff.	TAVR (n = 20,237)	SAVR (n = 20,237)	*p*-value	Std. diff.
Demographics									
Age		78.3 ± 8.6	66.6 ± 13.6	**<0.001**	**1.02**	73.3 ± 9.3	73.2 ± 8.5	0.37	0.009
Male		27,977 (55.5)	28,486 (63.8)	**<0.001**	**0.2**	12,297 (60.8)	12,292 (60.7)	0.96	0.001
White		43,346 (86.0)	28,168 (63.1)	**<0.001**	**0.5**	16,098 (79.5)	16,207 (80.1)	0.18	0.01
Black or African American		2490 (4.9)	2437 (5.5)	**<0.001**	0.02	1103 (5.5)	1111 (5.5)	0.86	0.002
Hispanic or Latino		1530 (3.0)	1544 (3.5)	**<0.001**	0.02	642 (3.2)	647 (3.2)	0.89	0.001
Nicotine dependence		15,860 (31.5)	7815 (17.5)	**<0.001**	**0.34**	5240 (25.9)	5158 (25.5)	0.35	0.009
Comorbidities									
Atrial Fibrillation		18,133 (36.0)	11,065 (24.8)	**<0.001**	**0.25**	6508 (32.2)	6544 (32.3)	0.70	0.004
Heart Failure		29,156 (57.8)	13,682 (30.7)	**<0.001**	**0.57**	8826 (43.6)	8913 (44.0)	0.38	0.009
COPD		10,293 (20.4)	4610 (10.3)	**<0.001**	**0.28**	3316 (16.4)	3241 (16.0)	0.31	0.01
Hypertension		38,438 (76.3)	24,736 (55.4)	**<0.001**	**0.45**	14,127 (69.8)	14,116 (69.8)	0.91	0.001
CKD		15,573 (30.9)	6216 (13.9)	**<0.001**	**0.42**	4455 (22.0)	4443 (22.0)	0.89	0.001
Peripheral Vascular Disease		7509 (14.9)	3049 (6.8)	**<0.001**	**0.26**	2287 (11.3)	2246 (11.1)	0.52	0.006
Atherosclerosis		14,863 (29.5)	5029 (11.3)	**<0.001**	**0.46**	3850 (19.0)	3792 (18.7)	0.46	0.007
IHD		38,034 (75.4)	25,267 (56.6)	**<0.001**	**0.41**	14,273 (70.5)	14,270 (70.5)	0.97	<0.001
Diabetes Mellitus		18,819 (37.3)	11,523 (25.8)	**<0.001**	**0.25**	7243 (35.8)	7210 (35.6)	0.73	0.003
Ischemic Stroke		3950 (7.8)	2370 (5.3)	**<0.001**	**0.10**	1379 (6.8)	1407 (7.0)	0.58	0.005
BAV		826 (1.6)	1802 (4.0)	**<0.001**	**0.15**	560 (2.8)	561 (2.8)	0.98	<0.001
Aortic Aneurysm		525 (1.0)	580 (1.3)	**<0.001**	0.02	208 (1.0)	211 (1.0)	0.88	0.001
Laboratory									
SBP (mmHg)		129.5 ± 21.1	127.5 ± 20.7	**<0.001**	0.09	129.1 ± 20.9	128.7 ± 21	0.12	0.02
LDL Cholesterol (mg/dL)		81.8 ± 34.4	91 ± 37.9	**<0.001**	**0.25**	84.2 ± 35.7	86.6 ± 36.3	**<0.001**	0.07
	≥160 mg/dL	2642 (5.2)	2178 (4.9)	**<0.001**	0.07	1144 (5.7)	1104 (5.5)	0.39	0.009
Hemoglobin A1c (%)		6.2 ± 1.2	6.0 ± 1.3	**<0.001**	**0.13**	6.2 ± 1.3	6.2 ± 1.3	0.81	0.003
BMI (kg/m^2^)		29.3 ± 6.8	29.7 ± 6.5	**<0.001**	0.06	30.2 ± 7.3	29.7 ± 6.2	**<0.001**	0.07
	≥30 kg/m^2^	22,472 (44.6)	15,006 (33.6)	**<0.001**	**0.23**	8737 (43.2)	8722 (43.1)	0.73	0.003
Creatinine (mg/dL)		1.3 ± 1.4	1.2 ± 1.1	**<0.001**	**0.1**	1.3 ± 1.9	1.2 ± 1.0	**<0.001**	0.08
	≥1.2 mg/dL	25,605 (50.8)	14,483 (32.5)	**<0.001**	**0.38**	8545 (42.2)	8365 (41.3)	0.67	0.004
LVEF (%)		55.8 ± 14.5	55.5 ± 14.7	0.29	0.02	55.7 ± 14.6	55.5 ± 14.9	0.62	0.01
aPTT		34 ± 13.2	33.2 ± 12.3	**<0.001**	0.07	33.8 ± 13.2	34.3 ± 13.6	**0.001**	0.04
	≥35 s	14,429 (28.6)	10,429 (23.4)	**<0.001**	**0.12**	5583 (27.6)	5586 (27.6)	0.973	<0.01
PT		13.9 ± 4.5	13.1 ± 3.7	**<0.001**	**0.19**	13.6 ± 4.3	13.4 ± 3.8	**<0.001**	0.05
	≥13.5 s	21,507 (42.7)	13,141 (29.4)	**<0.001**	**0.28**	7601 (37.6)	7650 (37.8)	0.62	0.005

Continuous variables are presented as mean ± SD. Categorical variables are presented as n (%). Bold values denote statistical significance. aPTT, activated partial thromboplastin time; BAV, bicuspid aortic valve; BMI, body mass index; CKD, chronic kidney disease; COPD, chronic obstructive pulmonary disease; LDL, low-density lipoprotein; LVEF, left ventricular ejection fraction; PSM, propensity score-matching; PT, prothrombin time; SBP, systolic blood pressure; SAVR, surgical aortic valve replacement; Std. diff., standardized mean difference; TAVR, transcatheter aortic valve replacement.

### 3.2 Outcomes

Despite low event counts, TAVR was associated with a significantly lower risk of composite AD compared to SAVR, and this association was consistent across all time points analyzed. At 1 year, the odds of composite AD were significantly lower in the TAVR group (OR 0.60; 95% CI 0.44–0.82; *p* < 0.01), and this remained consistent at 3 years (OR 0.64; 95% CI 0.48–0.83; *p* < 0.01) and 5 years (OR 0.66; 95% CI 0.51–0.85; *p* < 0.01).

**Fig. 2.**
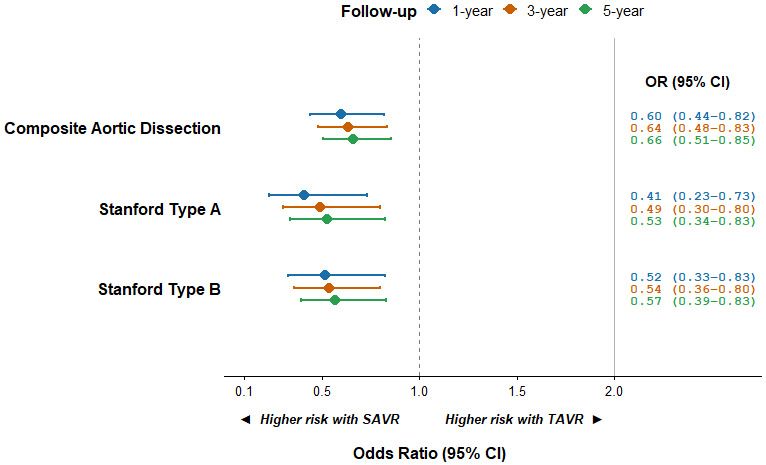
**Forest plot of odds ratios (OR) and 95% confidence intervals (CI) for aortic dissection outcomes at 1-year, 3-year, and 5-year follow-up, stratified by dissection type (Composite Aortic Dissection, Stanford Type A, and Stanford Type B)**. CI, confidence interval; OR, odds ratio; TAVR, transcatheter aortic valve replacement; SAVR, surgical aortic valve replacement.

When stratified by Stanford classification, TAVR was associated with significantly lower odds of both Type A and Type B AD at all time points. For Stanford Type A, the OR was 0.41 (95% CI 0.23–0.73) at 1 year, 0.49 (95% CI 0.30–0.80) at 3 years, and 0.53 (95% CI 0.34–0.83) at 5 years (all *p* < 0.01). For Stanford Type B, the OR was 0.52 (95% CI 0.33–0.83) at 1 year, 0.54 (95% CI 0.36–0.80) at 3 years, and 0.57 (95% CI 0.39–0.83) at 5 years (all *p* < 0.01). Full event counts and cumulative incidences are presented in Table 2, and effect size comparisons across all time points and AD types are shown in Fig. 2.

**Table 2.** **Incidence of aortic dissection events after TAVR vs SAVR at 1-, 3-, and 5-years stratified by stanford classification after propensity score-matching**.

Outcome	TAVR (n = 20,237)			SAVR (n = 20,237)		
	1-year	3-year	5-year	1-year	3-year	5-year
Composite aortic dissection	65 (0.32%)	84 (0.42%)	95 (0.47%)	108 (0.53%)	132 (0.65%)	144 (0.71%)
Stanford type A	16 (0.08%)	24 (0.12%)	29 (0.14%)	39 (0.19%)	49 (0.24%)	55 (0.27%)
Stanford type B	27 (0.13%)	37 (0.18%)	44 (0.22%)	52 (0.26%)	69 (0.34%)	77 (0.38%)

Events presented as n (cumulative incidence %). Follow-up truncated at 1, 3, and 5 years. SAVR, surgical aortic valve replacement; TAVR, transcatheter aortic valve replacement.

### 3.3 Time-to-Event Analysis

**Fig. 3.**
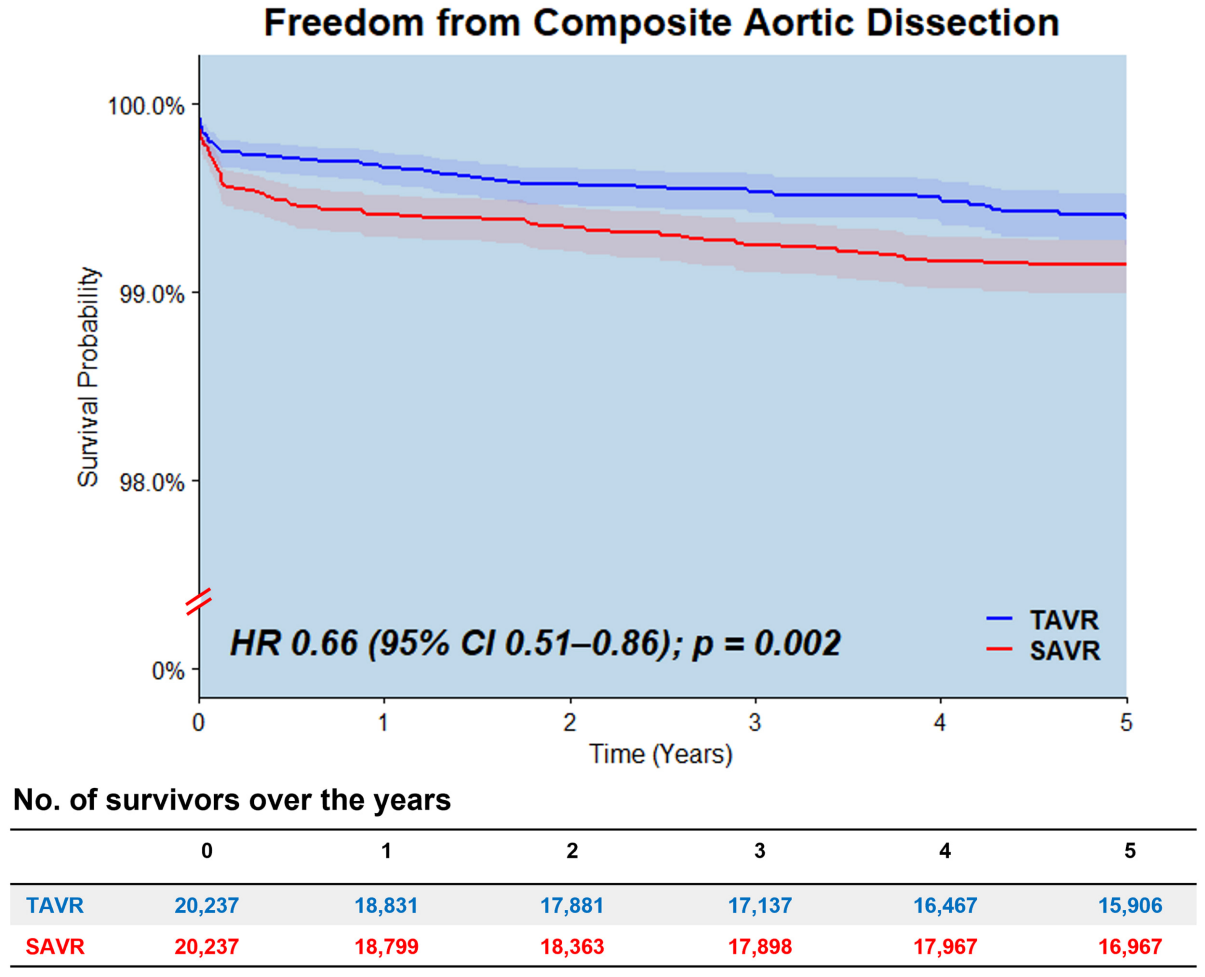
**Kaplan–Meier curves reporting freedom from composite aortic dissection across five years**. HR, Hazard Ratio; TAVR, transcatheter aortic valve replacement; SAVR, surgical aortic valve replacement.

KM curves demonstrated significantly greater freedom from AD in the TAVR group compared with the SAVR group over the 5-year follow-up period, with curves separating early and remaining consistently divergent throughout the observation window. Time to-event analysis confirmed a significantly lower hazard of composite AD in the TAVR group compared with SAVR (HR 0.66; 95% CI 0.51–0.86; *p* = 0.002; Fig. 3). When stratified by Stanford classification, TAVR was associated with a significantly lower hazard of both Type A AD (HR 0.53; 95% CI 0.34–0.84; *p* = 0.005; **Supplementary Fig. 1A**) and Type B AD (HR 0.58; 95% CI 0.39–0.84; *p* = 0.003; **Supplementary Fig. 1B**).

To assess the robustness of findings, multivariable Cox regression was performed in the unmatched cohort (n = 95,043), adjusting for the same covariates used in the PSM model. TAVR remained independently associated with a significantly lower hazard of composite AD (HR 0.69; 95% CI 0.56–0.85; *p* = 0.0004), Stanford Type A AD (HR 0.55; 95% CI 0.38–0.79; *p* = 0.002), and Stanford Type B AD (HR 0.66; 95% CI 0.49–0.88; *p* = 0.004) compared with SAVR. These findings were consistent with the primary matched analysis, supporting the robustness of the observed association. Full results are presented in Table 3.

**Table 3.** **Multivariable cox regression analysis**.

Outcome	Procedure	Adjusted HR	95% CI	*p*-value
Composite aortic dissection	TAVR	0.69	0.56–0.85	0.0004
	SAVR	1.45	1.18–1.77	
Stanford Type A	TAVR	0.55	0.38–0.79	0.002
	SAVR	1.82	1.26–2.65	
Stanford Type B	TAVR	0.66	0.49–0.88	0.004
	SAVR	1.52	1.13–2.02	

CI, confidence interval; HR, hazard ratio; SAVR, surgical aortic valve replacement; TAVR, transcatheter aortic valve replacement.

## 4. Discussion

In this large, real-world, PSM cohort study of patients undergoing isolated AVR for AVD, TAVR was associated with a significantly lower risk of AD compared with SAVR. This association was evident early and remained consistent over a 5-year follow-up. TAVR was associated with statistically significant lower odds and hazard of composite AD, and both Stanford Type A and Type B AD compared with SAVR.

Procedurally, lower rates of AD with TAVR are mechanistically plausible. SAVR involves aortic cross-clamping, cardioplegia, and direct aortic manipulation, all of which increase mechanical stress on the aortic wall and may precipitate AD [13,14,15], particularly in older patients or those with underlying medial degeneration [16,17]. In contrast, TAVR avoids sternotomy and direct aortic handling, thereby minimizing iatrogenic vascular injury [18]. Prior studies have shown that the aortic wall’s tensile strength declines with age, even in patients without diagnosed connective tissue disease, which may explain the increased vulnerability to AD during open surgery in this population [19,20,21].

Because AD is rare following AVR, prior comparisons between TAVR and SAVR for this complication have been limited. Small case series, case reports, and national procedural databases have documented the occurrence of AD following both procedures, but often without head-to-head comparison or long-term follow-up [10,22,23,24,25,26,27,28,29,30]. Our findings fill this gap, providing comparative incidence data at multiple intervals and strengthening the argument that procedural access and invasiveness are key modifiers of long-term vascular risk.

Our findings build upon and extend previous observational data, most notably the expert review by DeGraaff et al. [6], which highlights that the incidence of TAVR-related AD is low, typically ranging from 0.1% to 1.9% across major registries such as the German Aortic Valve Registry (GARY) and the European Registry of Transcatheter Aortic Valve Implantation (EuRECS-TAVI) [28,29]. In their analysis of over 27,000 TAVR patients in EuRECS-TAVI, only 0.1% experienced AD requiring surgical conversion. However, that analysis lacked a comparator group and focused predominantly on emergent Type A AD. In contrast, our study includes over 40,000 matched patients and demonstrates a statistically significantly lower composite AD rate following TAVR than SAVR (0.47% vs. 0.71%; OR 0.66; *p* < 0.01), with Stanford Type A (0.14% vs. 0.27%; OR 0.53; *p* < 0.01) and Type B (0.22 vs. 0.38%; OR 0.57; *p* < 0.01) stratification and a matched comparator arm and across a 5-year follow-up.

DeGraaff et al. [6] also emphasized the difficulty of detecting type B or anatomically distal AD after TAVR due to their often asymptomatic nature and underreporting in surgical registries. Our study addresses this limitation through Stanford Type A and Type B classification applied consistently across both cohorts, enabling a more complete capture of AD events regardless of anatomical location. Despite concerns about descending AD during catheter manipulation [30], TAVR was associated with a significantly lower hazard of both Type A and Type B AD compared with SAVR. Whether catheter-based access contributes to distal AD risk warrants further investigation in dedicated studies with granular access-site data.

The observed reduction in both Type A and Type B AD with TAVR suggests that the protective effect extends beyond avoidance of direct aortic incision alone and may reflect broader benefits of minimizing perioperative hemodynamic stress and aortic wall trauma across the entire aorta. One potential explanation is that ADs involving multiple regions or those localized to more distal, heavily atherosclerotic segments, such as the abdominal or more distal segments of the descending thoracic aorta, are more sensitive to systemic factors such as perioperative hemodynamic shifts and aortic wall fragility, possessing different biochemical properties and origin of vascular smooth muscle cells [31,32].

Prior reports have characterized the incidence of AD in the setting of cardiac surgery and acute aortic syndromes. In the Society of Thoracic Surgeons (STS) database, intraoperative or early postoperative AD occurred in 0.06% of cardiac surgical procedures and in 0.09% of isolated aortic valve operations [33]. Similarly, data from the International Registry of Acute Aortic Dissection (IRAD) identified iatrogenic ADs as 2.5% of all acute AD, with Type A accounting for the majority of iatrogenic cases [34]. These reports describe perioperative or acute clinical presentations, whereas the present study evaluates the cumulative incidence of any coded AD during longitudinal follow-up after isolated AVR.

The similar pre-matched numbers of TAVR and SAVR procedures in our cohort reflect well-documented shifts in clinical practice. Over the past decade, TAVR adoption has expanded rapidly across U.S. centers, now exceeding SAVR for aortic stenosis. Recent national and regional reports consistently show this steady replacement of SAVR by TAVR across a broad range of patient groups [35,36].

The implications for patient selection are important. While valve durability and anatomical considerations remain central to choosing TAVR vs. SAVR, our findings may suggest, that in patients with borderline aortic morphology, frailty, or a known predisposition to AD (e.g., chronic hypertension, advanced age, male sex, aortic aneurysms and connective tissue disorders) [37,38,39,40], TAVR may offer a safer vascular profile, especially when initial echocardiographic data shows features associated with increased risk of AD, such as intimal flap visualization, aortic root dilation, and increased aortic stiffness. Many ADs occur in patients with aortic dimensions below standard thresholds for surgery, underscoring the need for comprehensive risk assessment beyond diameter alone [3,41,42,43].

### Strengths and Limitations

This study has several strengths. First, as one of the largest real-world analyses to date comparing AD risk between TAVR and SAVR, the study used a well-curated, nationally representative dataset. The use of rigorous 1:1 PSM helped ensure balanced baseline characteristics, minimizing confounding. Second, outcomes were classified using Stanford Type A and Type B designations, enabling clinically meaningful differentiation of AD events across both cohorts. Third, the availability of long-term follow-up data enabled time-to-event analyses extending well beyond the perioperative period, with HRs reflecting sustained differences in risk between the two groups. Finally, our findings were enhanced by multivariable regression analysis and replicated in the unmatched cohort, supporting the robustness and consistency of the primary PSM results.

Important study limitations must be acknowledged. AD events were identified through diagnostic codes, which may result in under-reporting or misclassification. Imaging confirmation was not available, and clinical context (e.g., traumatic vs. spontaneous, acute vs. chronic) could not be determined from the available data. Additionally, the unavailability of detailed imaging data prevented stratified analysis or PSM by aortic measurements. Procedural details such as valve type, access route, operator experience, or technical complications were also not available. Even though TriNetX tracks patients longitudinally only within participating institutions, AD events treated elsewhere may be under-captured. Furthermore, despite careful matching, residual confounding from unmeasured variables as well as selection bias remains possible, as is the case in any observational study. Additionally, as a federated database, TriNetX does not permit derivation of traditional at-risk tables across institutions; accordingly, a formal Fine-Gray competing-risk analysis could not be performed. Furthermore, while this study reports results through 5 years, not all ADs occurring late in follow-up can be confidently attributed to the index AVR procedure. Some events may reflect underlying patient risk factors or age-related aortic degeneration rather than direct consequences of surgical or transcatheter intervention. Early TAVR recipients were often extreme- or high-risk patients, and differences in frailty or procedural selection that are not fully captured by coded variables may influence long-term outcomes. Due to platform limitations of TriNetX, the number of patients excluded for each criterion could not be determined, as only aggregate cohort selection steps are available.

Finally, despite the large overall cohort, absolute event counts for AD remain low, reflecting the inherent rarity of this complication following AVR. As such, odds ratios, hazard ratios, and the associated 95% confidence intervals may lack precision. Accordingly, these findings should be interpreted as hypothesis-generating and as a framework for future studies with detailed imaging and adjudicated outcomes.

## 5. Conclusion

In this large, real-world, PSM cohort study, despite low event rates, TAVR was associated with a significantly lower risk of AD compared with SAVR, with consistent findings across both Stanford Type A and Type B AD and over a 5-year follow-up. These results suggest that the less invasive nature of TAVR may be associated with a vascular safety advantage, particularly in patients at elevated risk for aortic wall complications. While absolute event rates remain low, the observed differences carry important implications for procedural planning and patient selection. Future studies incorporating imaging data and device-specific details may further clarify the mechanisms underlying these findings and guide individualized risk assessment.

## Data Availability

The data underlying this article will be shared on a reasonable request to the corresponding authors.

## Abbreviations

AD, aortic dissection; AVD, aortic valve disease; AVR, aortic valve replacement; CI, confidence interval; HR, hazard ratio; OR, odds ratio; PSM, propensity score-matching; SAVR, surgical aortic valve replacement; TAVR, transcatheter aortic valve replacement.
