# Supplementary material for: Transcatheter Aortic Valve Replacement is Associated With a Lower Risk of Aortic Dissection Than Surgical Aortic Valve Replacement: A Propensity Score-Matched Analysis
Source: Rev Cardiovasc Med. 2026 Jul 23;27(7):50574. doi: 10.31083/RCM50574 (PMC13419957; doi:10.31083/RCM50574)
Supplement: Supplementary file 1 [file 2153-8174-27-7-50574-s1.zip › Supplementary Material.docx]

**Transcatheter Aortic Valve Replacement is Associated with Lower Risk of Aortic Dissection Than Surgical Aortic Valve Replacement: A Propensity Score-Matched Analysis**

**Supplementary Material**

**Table of contents**

[**Supplementary Table 1.** STROBE Statement—Checklist of items that should be included in reports of cohort studies. 1](#_Toc227337914)

[**Supplementary Table 2.** Definition of baseline characteristics of the included population. 5](#_Toc227337915)

[**Supplementary Table 3.** Diagnosis and Procedural Codes Used to Define Cohorts’ inclusion and exclusion criteria. 8](#_Toc227337916)

**Supplementary Table 4.** Follow-up metrics at each time point after propensity score-matching.. 16

[**Supplementary Table 5.** Procedural and diagnostic codes used for outcome definition. 16](#_Toc227337917)

[**Supplementary Methods 1.** Details of Propensity Score Matching and Covariate Balance Metrics. 18](#_Toc227337918)

[**Supplementary Fig. 1**. Kaplan–Meier curves reporting freedom from aortic dissection according to Stanford classification. 20](#_Toc227337919)

# **Supplementary Table 1.** STROBE Statement—Checklist of items that should be included in reports of cohort studies.

|  | **Item No** | **Recommendation** | **Page No.** |
| --- | --- | --- | --- |
| **Title and abstract** | **1** | **(*a*) Indicate the study’s design with a commonly used term in the title or the abstract** | **1** |
|  |  | **(*b*) Provide in the abstract an informative and balanced summary of what was done and what was found** | **3** |
| **Introduction** | | |  |
| **Background/rationale** | **2** | **Explain the scientific background and rationale for the investigation being reported** | **5** |
| **Objectives** | **3** | **State specific objectives, including any prespecified hypotheses** | **6** |
| **Methods** | | |  |
| **Study design** | **4** | **Present key elements of study design early in the paper** | **7** |
| **Setting** | **5** | **Describe the setting, locations, and relevant dates, including periods of recruitment, exposure, follow-up, and data collection** | **7** |
| **Participants** | **6** | **(*a*) Give the eligibility criteria, and the sources and methods of selection of participants. Describe methods of follow-up** | **8** |
|  |  | **(*b*) For matched studies, give matching criteria and number of exposed and unexposed** | **8 & in the suppl.** |
| **Variables** | **7** | **Clearly define all outcomes, exposures, predictors, potential confounders, and effect modifiers. Give diagnostic criteria, if applicable** | **8** |
| **Data sources/ measurement** | **8*** | **For each variable of interest, give sources of data and details of methods of assessment (measurement). Describe comparability of assessment methods if there is more than one group** | ***7*** |
| **Bias** | **9** | **Describe any efforts to address potential sources of bias** | **8** |
| **Study size** | **10** | **Explain how the study size was arrived at** | **Fig 1** |
| **Quantitative variables** | **11** | **Explain how quantitative variables were handled in the analyses. If applicable, describe which groupings were chosen and why** | **8** |
| **Statistical methods** | **12** | **(*a*) Describe all statistical methods, including those used to control for confounding** | **8** |
|  |  | **(*b*) Describe any methods used to examine subgroups and interactions** | **8** |
|  |  | **(*c*) Explain how missing data were addressed** | **8** |
|  |  | **(*d*) If applicable, explain how loss to follow-up was addressed** | **8** |
|  |  | **(*e*) Describe any sensitivity analyses** | **8** |
| **Results** | | |  |
| **Participants** | **13*** | **(a) Report numbers of individuals at each stage of study—eg numbers potentially eligible, examined for eligibility, confirmed eligible, included in the study, completing follow-up, and analysed** | **9** |
|  |  | **(b) Give reasons for non-participation at each stage** | **Fig 1.** |
|  |  | **(c) Consider use of a flow diagram** |  |
| **Descriptive data** | **14*** | **(a) Give characteristics of study participants (eg demographic, clinical, social) and information on exposures and potential confounders** | **Table 1.** |
|  |  | **(b) Indicate number of participants with missing data for each variable of interest** | **-** |
|  |  | **(c) Summarise follow-up time (eg, average and total amount)** | **9** |
| **Outcome data** | **15*** | **Report numbers of outcome events or summary measures over time** | **Table 2.** |
| **Main results** | **16** | **(*a*) Give unadjusted estimates and, if applicable, confounder-adjusted estimates and their precision (eg, 95% confidence interval). Make clear which confounders were adjusted for and why they were included** | **Table 2.** |
|  |  | **(*b*) Report category boundaries when continuous variables were categorized** | **9-10** |
|  |  | **(*c*) If relevant, consider translating estimates of relative risk into absolute risk for a meaningful time period** | **9-10** |
| **Other analyses** | **17** | **Report other analyses done—eg analyses of subgroups and interactions, and sensitivity analyses** | **9-10** |
| **Discussion** | | |  |
| **Key results** | **18** | **Summarise key results with reference to study objectives** | **11** |
| **Limitations** | **19** | **Discuss limitations of the study, taking into account sources of potential bias or imprecision. Discuss both direction and magnitude of any potential bias** | **13** |
| **Interpretation** | **20** | **Give a cautious overall interpretation of results considering objectives, limitations, multiplicity of analyses, results from similar studies, and other relevant evidence** | **12** |
| **Generalisability** | **21** | **Discuss the generalisability (external validity) of the study results** | **12** |
| **Other information** | | |  |
| **Funding** | **22** | **Give the source of funding and the role of the funders for the present study and, if applicable, for the original study on which the present article is based** | **1** |

# **Supplementary Table 2.** Definition of baseline characteristics of the included population.

| **Diagnosis** | |
| --- | --- |
| **Code** | **Characteristic** |
| AI | Age at Index |
| M | Male |
| 2106-3 | White |
| 2054-5 | Black or African American |
| 2135-2 | Hispanic or Latino |
| Z87.891 | Personal history of nicotine dependence |
| **Diagnosis** | |
| I48 | Atrial fibrillation and flutter |
| I50 | Heart failure |
| J44 | Other chronic obstructive pulmonary disease |
| I10 | Essential (primary) hypertension |
| N18 | Chronic kidney disease (CKD) |
| I73 | Other peripheral vascular diseases |
| I70 | Atherosclerosis |
| I20-I25 | Ischemic heart diseases |
| E08-E13 | Diabetes mellitus |
| I63 | Cerebral infarction |
| Q23.81 | Bicuspid aortic valve |
| I71.9 | Aortic aneurysm of unspecified site, without rupture |
| I48 | Atrial fibrillation and flutter |
| I50 | Heart failure |
| J44 | Other chronic obstructive pulmonary disease |
| I10 | Essential (primary) hypertension |
| N18 | Chronic kidney disease (CKD) |
| I73 | Other peripheral vascular diseases |
| I70 | Atherosclerosis |
| **Laboratory** | |
| 9085 | Blood Pressure, Systolic |
| 9002 | Cholesterol in LDL [Mass/volume] in Serum or Plasma |
| 9037 | Hemoglobin A1c/Hemoglobin.total in Blood |
| 9083 | BMI |
| 9024 | Creatinine [Mass/volume] in Serum, Plasma or Blood |
| 2003 | Left Ventricular Ejection Fraction (LVEF) (%) |
| 9031 | Activated partial thromboplastin time (aPTT) in Plasma or Blood |
| 9033 | Prothrombin time (PT) in Plasma or Blood |

# **Supplementary Table 3.** Diagnosis and Procedural Codes Used to Define Cohorts’ inclusion and exclusion criteria.

**Supplementary Table 3A.** Diagnosis and Procedural Codes for Cohort 1 (TAVR).

|  | | | | | |
| --- | --- | --- | --- | --- | --- |
|  | | | | | |
|  | must have |  | demographics | Age | Age (at least 18 years (most recent occurrence)) |
|  |  | and any of | demographics | UMLS:HL7V3.0:Gender:M | Male |
|  |  |  | demographics | UMLS:HL7V3.0:Gender:F | Female |
| Group 1 | | | | | |
|  | **Aortic valve disorders** | | | | |
|  | must have | any of | diagnosis | UMLS:ICD10CM:I35.0 | Nonrheumatic aortic (valve) stenosis |
|  |  |  | diagnosis | UMLS:ICD10CM:I35.1 | Nonrheumatic aortic (valve) insufficiency |
|  |  |  | diagnosis | UMLS:ICD10CM:I35.2 | Nonrheumatic aortic (valve) stenosis with insufficiency |
|  |  |  | diagnosis | UMLS:ICD10CM:I35.8 | Other nonrheumatic aortic valve disorders |
|  |  |  | diagnosis | UMLS:ICD10CM:I35.9 | Nonrheumatic aortic valve disorder, unspecified |
| Group 2 | | | | | |
|  | **Group 2A TAVR** | | | | |
|  | must have | any of | procedure | UMLS:ICD10PCS:02RF3JZ | Replacement of Aortic Valve with Synthetic Substitute, Percutaneous Approach |
|  |  |  | procedure | UMLS:ICD10PCS:02RF37Z | Replacement of Aortic Valve with Autologous Tissue Substitute, Percutaneous Approach |
|  |  |  | procedure | UMLS:ICD10PCS:02WA3JZ | Revision of Synthetic Substitute in Heart, Percutaneous Approach |
|  |  |  | procedure | UMLS:ICD10PCS:02RF38Z | Replacement of Aortic Valve with Zooplastic Tissue, Percutaneous Approach |
|  |  |  | procedure | UMLS:CPT:33361 | Transcatheter aortic valve replacement (TAVR/TAVI) with prosthetic valve; percutaneous femoral artery approach |
|  |  |  | procedure | UMLS:CPT:33362 | Transcatheter aortic valve replacement (TAVR/TAVI) with prosthetic valve; open femoral artery approach |
|  |  |  | procedure | UMLS:CPT:33363 | Transcatheter aortic valve replacement (TAVR/TAVI) with prosthetic valve; open axillary artery approach |
|  |  |  | procedure | UMLS:CPT:33364 | Transcatheter aortic valve replacement (TAVR/TAVI) with prosthetic valve; open iliac artery approach |
|  |  |  | procedure | UMLS:CPT:33365 | Transcatheter aortic valve replacement (TAVR/TAVI) with prosthetic valve; transaortic approach (eg, median sternotomy, mediastinotomy) |
|  |  |  | procedure | UMLS:CPT:33366 | Transcatheter aortic valve replacement (TAVR/TAVI) with prosthetic valve; transapical exposure (eg, left thoracotomy) |
|  |  |  | procedure | UMLS:CPT:33367 | Transcatheter aortic valve replacement (TAVR/TAVI) with prosthetic valve; cardiopulmonary bypass support with percutaneous peripheral arterial and venous cannulation (eg, femoral vessels) (List separately in addition to code for primary procedure) |
|  |  |  | procedure | UMLS:CPT:33368 | Transcatheter aortic valve replacement (TAVR/TAVI) with prosthetic valve; cardiopulmonary bypass support with open peripheral arterial and venous cannulation (eg, femoral, iliac, axillary vessels) (List separately in addition to code for primary procedure) |
|  |  |  | procedure | UMLS:CPT:33369 | Transcatheter aortic valve replacement (TAVR/TAVI) with prosthetic valve; cardiopulmonary bypass support with central arterial and venous cannulation (eg, aorta, right atrium, pulmonary artery) (List separately in addition to code for primary procedure) |
|  | date constraint | | The terms in this group occurred between Jan 1, 2012 and Dec 31, 2022 | | |
|  | event relationship | | Any instance of Previous or concomittant surgeries occurred on or before any instance of TAVR | | |
|  | **Group 2B Previous or concomittant surgeries** | | | | |
|  | cannot have |  | procedure | UMLS:ICD10PCS:02BW0ZZ | Excision of Thoracic Aorta, Descending, Open Approach |
|  |  | or | procedure | UMLS:ICD10PCS:02QW0ZZ | Repair Thoracic Aorta, Descending, Open Approach |
|  |  | or | procedure | UMLS:ICD10PCS:02RW0JZ | Replacement of Thoracic Aorta, Descending with Synthetic Substitute, Open Approach |
|  |  | or | procedure | UMLS:ICD10PCS:02UW0JZ | Supplement Thoracic Aorta, Descending with Synthetic Substitute, Open Approach |
|  |  | or | procedure | UMLS:CPT:33860 | Ascending aorta graft, with cardiopulmonary bypass, includes valve suspension, when performed (deprecated 2021) |
|  |  | or | procedure | UMLS:CPT:1035651 | Ascending aorta graft, with cardiopulmonary bypass, includes valve suspension, when performed |
|  |  | or | procedure | UMLS:CPT:33863 | Ascending aorta graft, with cardiopulmonary bypass, with aortic root replacement using valved conduit and coronary reconstruction (eg, Bentall) |
|  |  | or | procedure | UMLS:CPT:33864 | Ascending aorta graft, with cardiopulmonary bypass with valve suspension, with coronary reconstruction and valve-sparing aortic root remodeling (eg, David Procedure, Yacoub Procedure) |
|  |  | or | procedure | UMLS:SNOMED:312311004 | Replacement of ascending aorta |
|  |  | or | procedure | UMLS:SNOMED:699279007 | Aortic arch replacement |
|  |  | or | procedure | UMLS:CPT:33870 | Transverse arch graft, with cardiopulmonary bypass (deprecated 2021) |
|  |  | or | procedure | UMLS:CPT:1006313 | Endovascular Repair Procedures of the Descending Thoracic Aorta |
|  |  | or | procedure | UMLS:CPT:1006306 | Repair Procedures for Thoracic Aortic Aneurysm |
|  |  | or | procedure | UMLS:CPT:1006288 | Repair Procedures for Aortic Anomalies |
|  |  | or | procedure | UMLS:CPT:33877 | Repair of thoracoabdominal aortic aneurysm with graft, with or without cardiopulmonary bypass |
|  |  | or | procedure | UMLS:CPT:33875 | Descending thoracic aorta graft, with or without bypass |
|  |  | or | procedure | UMLS:CPT:35081 | Direct repair of aneurysm, pseudoaneurysm, or excision (partial or total) and graft insertion, with or without patch graft; for aneurysm, pseudoaneurysm, and associated occlusive disease, abdominal aorta |
|  |  | or | procedure | UMLS:CPT:35082 | Direct repair of aneurysm, pseudoaneurysm, or excision (partial or total) and graft insertion, with or without patch graft; for ruptured aneurysm, abdominal aorta |
|  |  | or | procedure | UMLS:CPT:35091 | Direct repair of aneurysm, pseudoaneurysm, or excision (partial or total) and graft insertion, with or without patch graft; for aneurysm, pseudoaneurysm, and associated occlusive disease, abdominal aorta involving visceral vessels (mesenteric, celiac, renal) |
|  |  | or | procedure | UMLS:CPT:1031040 | Endovascular repair of infrarenal aorta by deployment of an aorto-aortic tube endograft including pre-procedure sizing and device selection, all nonselective catheterization(s), all associated radiological supervision and interpretation, all endograft extension(s) placed in the aorta from the level of the renal arteries to the aortic bifurcation, and all angioplasty/stenting performed from the level of the renal arteries to the aortic bifurcation |
|  |  | or | procedure | UMLS:CPT:1031042 | Endovascular repair of infrarenal aorta and/or iliac artery(ies) by deployment of an aorto-bi-iliac endograft including pre-procedure sizing and device selection, all nonselective catheterization(s), all associated radiological supervision and interpretation, all endograft extension(s) placed in the aorta from the level of the renal arteries to the iliac bifurcation, and all angioplasty/stenting performed from the level of the renal arteries to the iliac bifurcation |
|  |  | or | procedure | UMLS:CPT:1031041 | Endovascular repair of infrarenal aorta and/or iliac artery(ies) by deployment of an aorto-uni-iliac endograft including pre-procedure sizing and device selection, all nonselective catheterization(s), all associated radiological supervision and interpretation, all endograft extension(s) placed in the aorta from the level of the renal arteries to the iliac bifurcation, and all angioplasty/stenting performed from the level of the renal arteries to the iliac bifurcation |
|  |  | or | procedure | UMLS:SNOMED:76987002 | Aortoplasty |
|  |  | or | procedure | UMLS:SNOMED:232847003 | Aortic valve replacement and aortoplasty |
|  |  | or | procedure | UMLS:ICD10PCS:02RF08Z | Replacement of Aortic Valve with Zooplastic Tissue, Open Approach |
|  |  | or | procedure | UMLS:ICD10PCS:02QF0ZZ | Repair Aortic Valve, Open Approach |
|  |  | or | procedure | UMLS:ICD10PCS:02RF07Z | Replacement of Aortic Valve with Autologous Tissue Substitute, Open Approach |
|  |  | or | procedure | UMLS:ICD10PCS:02RF0JZ | Replacement of Aortic Valve with Synthetic Substitute, Open Approach |
|  |  | or | procedure | UMLS:ICD10PCS:02WA0JZ | Revision of Synthetic Substitute in Heart, Open Approach |
|  |  | or | procedure | UMLS:ICD10PCS:02WA0JZ | Revision of Synthetic Substitute in Heart, Open Approach |
|  |  | or | procedure | UMLS:ICD10PCS:02RF0KZ | Replacement of Aortic Valve with Nonautologous Tissue Substitute, Open Approach |
|  |  | or | procedure | UMLS:CPT:33405 | Replacement, aortic valve, open, with cardiopulmonary bypass; with prosthetic valve other than homograft or stentless valve |
|  |  | or | procedure | UMLS:CPT:33406 | Replacement, aortic valve, open, with cardiopulmonary bypass; with allograft valve (freehand) |
|  |  | or | procedure | UMLS:CPT:33410 | Replacement, aortic valve, open, with cardiopulmonary bypass; with stentless tissue valve |
| Group 3 | | | | | |
|  | **Connective Tissue Disease** | | | | |
|  | cannot have |  | diagnosis | UMLS:ICD10CM:Q79.6 | Ehlers-Danlos syndromes |
|  |  | or | diagnosis | UMLS:ICD10CM:Q87.41 | Marfan syndrome with cardiovascular manifestations |
|  |  | or | diagnosis | UMLS:ICD10CM:Q25.43 | Congenital aneurysm of aorta |
|  |  | or | diagnosis | UMLS:ICD10CM:I77.9 | Disorder of arteries and arterioles, unspecified |
|  |  | or | diagnosis | UMLS:ICD10CM:I77.6 | Arteritis, unspecified |
|  |  | or | diagnosis | UMLS:ICD10CM:Q96 | Turner's syndrome |

**Supplementary Table 3B.** Diagnosis and Procedural Codes for Cohort 2 (SAVR).

|  | | | | | |
| --- | --- | --- | --- | --- | --- |
|  | must have |  | demographics | Age | Age (at least 18 years (most recent occurrence)) |
|  |  | and any of | demographics | UMLS:HL7V3.0:Gender:M | Male |
|  |  |  | demographics | UMLS:HL7V3.0:Gender:F | Female |
| Group 1 | | | | | |
|  | **Aortic valve disorders** | | | | |
|  | must have | any of | diagnosis | UMLS:ICD10CM:I35.0 | Nonrheumatic aortic (valve) stenosis |
|  |  |  | diagnosis | UMLS:ICD10CM:I35.1 | Nonrheumatic aortic (valve) insufficiency |
|  |  |  | diagnosis | UMLS:ICD10CM:I35.2 | Nonrheumatic aortic (valve) stenosis with insufficiency |
|  |  |  | diagnosis | UMLS:ICD10CM:I35.8 | Other nonrheumatic aortic valve disorders |
|  |  |  | diagnosis | UMLS:ICD10CM:I35.9 | Nonrheumatic aortic valve disorder, unspecified |
| Group 2 | | | | | |
|  | **Group 2A SAVR** | | | | |
|  | must have | any of | procedure | UMLS:ICD10PCS:02RF08Z | Replacement of Aortic Valve with Zooplastic Tissue, Open Approach |
|  |  |  | procedure | UMLS:ICD10PCS:02QF0ZZ | Repair Aortic Valve, Open Approach |
|  |  |  | procedure | UMLS:ICD10PCS:02RF07Z | Replacement of Aortic Valve with Autologous Tissue Substitute, Open Approach |
|  |  |  | procedure | UMLS:ICD10PCS:02RF0JZ | Replacement of Aortic Valve with Synthetic Substitute, Open Approach |
|  |  |  | procedure | UMLS:ICD10PCS:02WA0JZ | Revision of Synthetic Substitute in Heart, Open Approach |
|  |  |  | procedure | UMLS:CPT:33405 | Replacement, aortic valve, open, with cardiopulmonary bypass; with prosthetic valve other than homograft or stentless valve |
|  |  |  | procedure | UMLS:CPT:33410 | Replacement, aortic valve, open, with cardiopulmonary bypass; with stentless tissue valve |
|  |  |  | procedure | UMLS:ICD10PCS:02RF0KZ | Replacement of Aortic Valve with Nonautologous Tissue Substitute, Open Approach |
|  |  |  | procedure | UMLS:CPT:33406 | Replacement, aortic valve, open, with cardiopulmonary bypass; with allograft valve (freehand) |
|  |  |  | procedure | UMLS:ICD10PCS:02WA0JZ | Revision of Synthetic Substitute in Heart, Open Approach |
|  | date constraint | | The terms in this group occurred between Jan 1, 2012 and Dec 31, 2022 | | |
|  | event relationship | | Any instance of Previous or concomittant surgeries to the aorta occurred on or before any instance of SAVR | | |
|  | **Group 2B Previous or concomittant surgeries to the aorta** | | | | |
|  | cannot have |  | procedure | UMLS:ICD10PCS:02BW0ZZ | Excision of Thoracic Aorta, Descending, Open Approach |
|  |  | or | procedure | UMLS:ICD10PCS:02QW0ZZ | Repair Thoracic Aorta, Descending, Open Approach |
|  |  | or | procedure | UMLS:ICD10PCS:02RW0JZ | Replacement of Thoracic Aorta, Descending with Synthetic Substitute, Open Approach |
|  |  | or | procedure | UMLS:ICD10PCS:02UW0JZ | Supplement Thoracic Aorta, Descending with Synthetic Substitute, Open Approach |
|  |  | or | procedure | UMLS:CPT:33860 | Ascending aorta graft, with cardiopulmonary bypass, includes valve suspension, when performed (deprecated 2021) |
|  |  | or | procedure | UMLS:CPT:1035651 | Ascending aorta graft, with cardiopulmonary bypass, includes valve suspension, when performed |
|  |  | or | procedure | UMLS:CPT:33863 | Ascending aorta graft, with cardiopulmonary bypass, with aortic root replacement using valved conduit and coronary reconstruction (eg, Bentall) |
|  |  | or | procedure | UMLS:CPT:33864 | Ascending aorta graft, with cardiopulmonary bypass with valve suspension, with coronary reconstruction and valve-sparing aortic root remodeling (eg, David Procedure, Yacoub Procedure) |
|  |  | or | procedure | UMLS:SNOMED:312311004 | Replacement of ascending aorta |
|  |  | or | procedure | UMLS:SNOMED:699279007 | Aortic arch replacement |
|  |  | or | procedure | UMLS:CPT:33870 | Transverse arch graft, with cardiopulmonary bypass (deprecated 2021) |
|  |  | or | procedure | UMLS:CPT:1006313 | Endovascular Repair Procedures of the Descending Thoracic Aorta |
|  |  | or | procedure | UMLS:CPT:1006306 | Repair Procedures for Thoracic Aortic Aneurysm |
|  |  | or | procedure | UMLS:CPT:1006288 | Repair Procedures for Aortic Anomalies |
|  |  | or | procedure | UMLS:CPT:33877 | Repair of thoracoabdominal aortic aneurysm with graft, with or without cardiopulmonary bypass |
|  |  | or | procedure | UMLS:CPT:33875 | Descending thoracic aorta graft, with or without bypass |
|  |  | or | procedure | UMLS:CPT:35081 | Direct repair of aneurysm, pseudoaneurysm, or excision (partial or total) and graft insertion, with or without patch graft; for aneurysm, pseudoaneurysm, and associated occlusive disease, abdominal aorta |
|  |  | or | procedure | UMLS:CPT:35082 | Direct repair of aneurysm, pseudoaneurysm, or excision (partial or total) and graft insertion, with or without patch graft; for ruptured aneurysm, abdominal aorta |
|  |  | or | procedure | UMLS:CPT:35091 | Direct repair of aneurysm, pseudoaneurysm, or excision (partial or total) and graft insertion, with or without patch graft; for aneurysm, pseudoaneurysm, and associated occlusive disease, abdominal aorta involving visceral vessels (mesenteric, celiac, renal) |
|  |  | or | procedure | UMLS:CPT:1031040 | Endovascular repair of infrarenal aorta by deployment of an aorto-aortic tube endograft including pre-procedure sizing and device selection, all nonselective catheterization(s), all associated radiological supervision and interpretation, all endograft extension(s) placed in the aorta from the level of the renal arteries to the aortic bifurcation, and all angioplasty/stenting performed from the level of the renal arteries to the aortic bifurcation |
|  |  | or | procedure | UMLS:CPT:1031041 | Endovascular repair of infrarenal aorta and/or iliac artery(ies) by deployment of an aorto-uni-iliac endograft including pre-procedure sizing and device selection, all nonselective catheterization(s), all associated radiological supervision and interpretation, all endograft extension(s) placed in the aorta from the level of the renal arteries to the iliac bifurcation, and all angioplasty/stenting performed from the level of the renal arteries to the iliac bifurcation |
|  |  | or | procedure | UMLS:CPT:1031042 | Endovascular repair of infrarenal aorta and/or iliac artery(ies) by deployment of an aorto-bi-iliac endograft including pre-procedure sizing and device selection, all nonselective catheterization(s), all associated radiological supervision and interpretation, all endograft extension(s) placed in the aorta from the level of the renal arteries to the iliac bifurcation, and all angioplasty/stenting performed from the level of the renal arteries to the iliac bifurcation |
|  |  | or | procedure | UMLS:SNOMED:76987002 | Aortoplasty |
|  |  | or | procedure | UMLS:SNOMED:232847003 | Aortic valve replacement and aortoplasty |
|  |  | or | procedure | UMLS:ICD10PCS:02RF3JZ | Replacement of Aortic Valve with Synthetic Substitute, Percutaneous Approach |
|  |  | or | procedure | UMLS:ICD10PCS:02WA3JZ | Revision of Synthetic Substitute in Heart, Percutaneous Approach |
|  |  | or | procedure | UMLS:CPT:33362 | Transcatheter aortic valve replacement (TAVR/TAVI) with prosthetic valve; open femoral artery approach |
|  |  | or | procedure | UMLS:ICD10PCS:02RF38Z | Replacement of Aortic Valve with Zooplastic Tissue, Percutaneous Approach |
|  |  | or | procedure | UMLS:ICD10PCS:02RF37Z | Replacement of Aortic Valve with Autologous Tissue Substitute, Percutaneous Approach |
|  |  | or | procedure | UMLS:CPT:33361 | Transcatheter aortic valve replacement (TAVR/TAVI) with prosthetic valve; percutaneous femoral artery approach |
|  |  | or | procedure | UMLS:CPT:33364 | Transcatheter aortic valve replacement (TAVR/TAVI) with prosthetic valve; open iliac artery approach |
|  |  | or | procedure | UMLS:CPT:33366 | Transcatheter aortic valve replacement (TAVR/TAVI) with prosthetic valve; transapical exposure (eg, left thoracotomy) |
|  |  | or | procedure | UMLS:CPT:33367 | Transcatheter aortic valve replacement (TAVR/TAVI) with prosthetic valve; cardiopulmonary bypass support with percutaneous peripheral arterial and venous cannulation (eg, femoral vessels) (List separately in addition to code for primary procedure) |
|  |  | or | procedure | UMLS:CPT:33365 | Transcatheter aortic valve replacement (TAVR/TAVI) with prosthetic valve; transaortic approach (eg, median sternotomy, mediastinotomy) |
|  |  | or | procedure | UMLS:CPT:33368 | Transcatheter aortic valve replacement (TAVR/TAVI) with prosthetic valve; cardiopulmonary bypass support with open peripheral arterial and venous cannulation (eg, femoral, iliac, axillary vessels) (List separately in addition to code for primary procedure) |
|  |  | or | procedure | UMLS:CPT:33369 | Transcatheter aortic valve replacement (TAVR/TAVI) with prosthetic valve; cardiopulmonary bypass support with central arterial and venous cannulation (eg, aorta, right atrium, pulmonary artery) (List separately in addition to code for primary procedure) |
|  |  | or | procedure | UMLS:CPT:33363 | Transcatheter aortic valve replacement (TAVR/TAVI) with prosthetic valve; open axillary artery approach |
| Group 3 | | | | | |
|  | **Connective Tissue and congenital diseases** | | | | |
|  | cannot have |  | diagnosis | UMLS:ICD10CM:Q79.6 | Ehlers-Danlos syndromes |
|  |  | or | diagnosis | UMLS:ICD10CM:Q87.41 | Marfan syndrome with cardiovascular manifestations |
|  |  | or | diagnosis | UMLS:ICD10CM:Q25.43 | Congenital aneurysm of aorta |
|  |  | or | diagnosis | UMLS:ICD10CM:I77.6 | Arteritis, unspecified |
|  |  | or | diagnosis | UMLS:ICD10CM:I77.8 | Other specified disorders of arteries and arterioles |
|  |  | or | diagnosis | UMLS:ICD10CM:Q96 | Turner's syndrome |

# **Supplementary Table 4.** Follow-up metrics at each time point after propensity score-matching.

**Supplementary Table 4A.** One-year follow-up metrics.

| **Follow-up Time** | | | | | | |
| --- | --- | --- | --- | --- | --- | --- |
|  |  | Cohort | Mean Follow-up (Days) | Standard Deviation | Median Follow-up (Days) | Interquartile Range |
|  |  | TAVR | 324.944 | 103.159 | 365 | 0 |
|  |  | SAVR | 297.921 | 129.164 | 365 | 0 |

**Supplementary Table 4B.** Three-year follow-up metrics.

| **Follow-up Time** | | | | | | |
| --- | --- | --- | --- | --- | --- | --- |
|  |  | Cohort | Mean Follow-up (Days) | Standard Deviation | Median Follow-up (Days) | Interquartile Range |
|  |  | TAVR | 831.580 | 382.218 | 1095 | 579 |
|  |  | SAVR | 816.241 | 431.938 | 1095 | 643 |

**Supplementary Table 4C.** Five-year follow-up metrics.

| **Follow-up Time** | | | | | | |
| --- | --- | --- | --- | --- | --- | --- |
|  |  | Cohort | Mean Follow-up (Days) | Standard Deviation | Median Follow-up (Days) | Interquartile Range |
|  |  | TAVR | 1142.353 | 642.438 | 1279 | 1309 |
|  |  | SAVR | 1224.406 | 726.967 | 1686 | 1373 |

# **Supplementary Table 5.** Procedural and diagnostic codes used for outcome definition.

| Composite | | | | |
| --- | --- | --- | --- | --- |
|  | **Outcome definition** | | | |
|  | | Diagnosis | UMLS:ICD10CM:I71.00 | Dissection of unspecified site of aorta |
|  | | Diagnosis | UMLS:ICD10CM:I71.01 | Dissection of thoracic aorta |
|  | | Diagnosis | UMLS:ICD10CM:I71.02 | Dissection of abdominal aorta |
|  | | Diagnosis | UMLS:ICD10CM:I71.03 | Dissection of thoracoabdominal aorta |
|  | | Diagnosis | UMLS:ICD9CM:441.0 | Dissecting aneurysm of aorta |
| Stanford Type A | | | | |
|  | **Outcome definition** | | | |
|  | | Diagnosis | UMLS:ICD10CM:I71.010 | Dissection of ascending aorta |
|  | | Diagnosis | UMLS:ICD10CM:I71.011 | Dissection of aortic arch |
|  | | Diagnosis | UMLS:ICD10CM:I71.012 | Dissection of descending thoracic aorta |
|  | | Diagnosis | UMLS:ICD10CM:I71.019 | Dissection of thoracic aorta, unspecified |
|  | | Diagnosis | UMLS:ICD9CM:441.01 | Dissection of aorta, thoracic |
| Stanford Type B | | | | |
|  | **Outcome definition** | | | |
|  | | Diagnosis | UMLS:ICD10CM:I71.012 | Dissection of descending thoracic aorta |
|  | | Diagnosis | UMLS:ICD10CM:I71.02 | Dissection of abdominal aorta |
|  | | Diagnosis | UMLS:ICD10CM:I71.03 | Dissection of thoracoabdominal aorta |
|  | | Diagnosis | UMLS:ICD10CM:I71.019 | Dissection of thoracic aorta, unspecified |
|  | | Diagnosis | UMLS:ICD9CM:441.01 | Dissection of aorta, thoracic |
| death | | | | |
|  | **Outcome definition** | | | |
|  | | Demographics | Deceased | Deceased |

# **Supplementary Methods 1.** Details of Propensity Score Matching and Covariate Balance Metrics.

| **Cohort 1 and cohort 2 patient count before and after propensity score matching** | | | | | |
| --- | --- | --- | --- | --- | --- |
|  | | Cohort | Patient count before matching | | Patient count after matching |
|  | | 1 - TAVR | 50,410 | | 20,237 |
|  | | 2 - SAVR | 44,633 | | 20,237 |
| **Propensity score density function - Before and after matching (cohort 1 - purple, cohort 2 - green)** | | | | | |
| 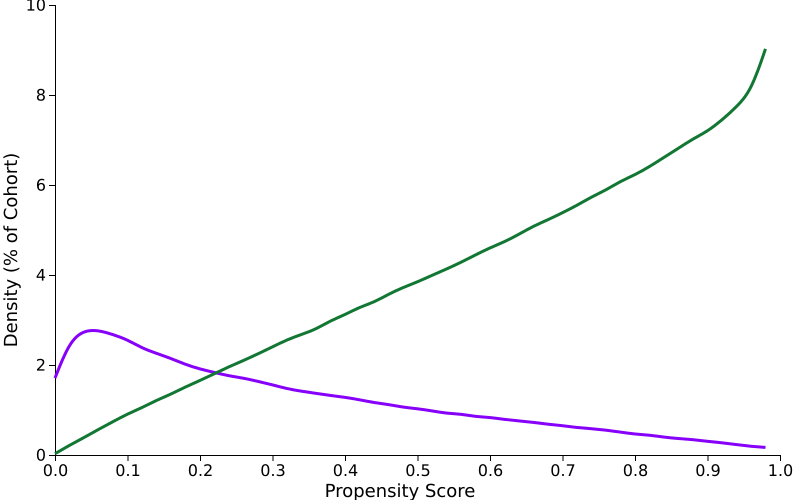 | 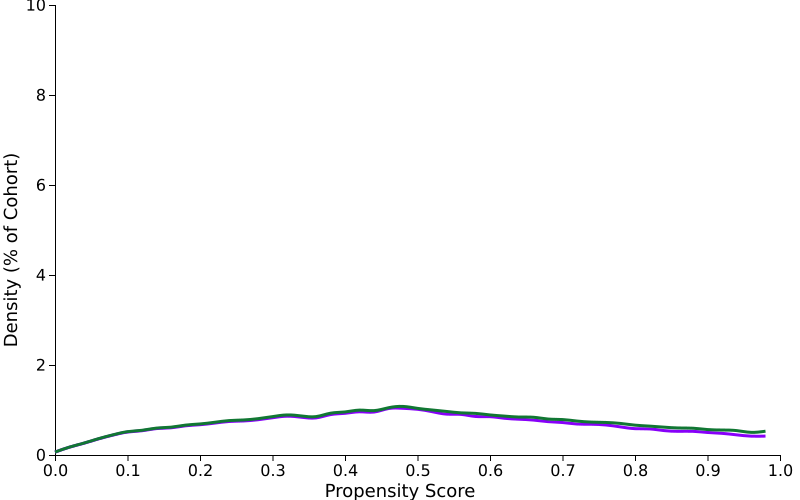 | 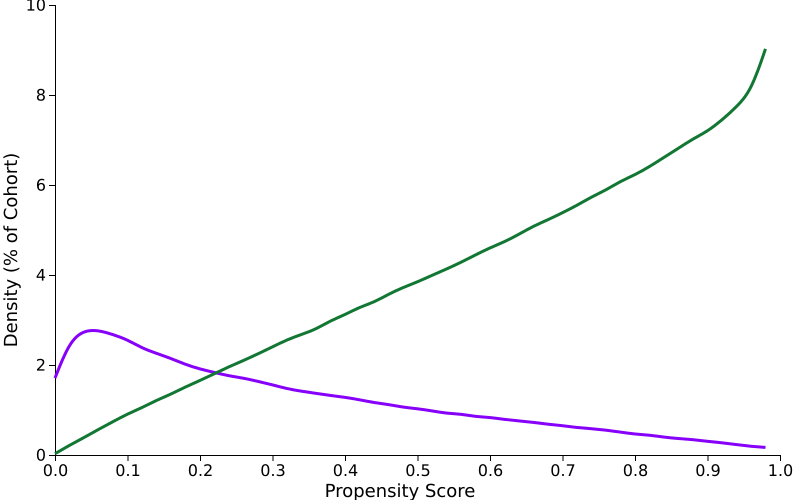 | | 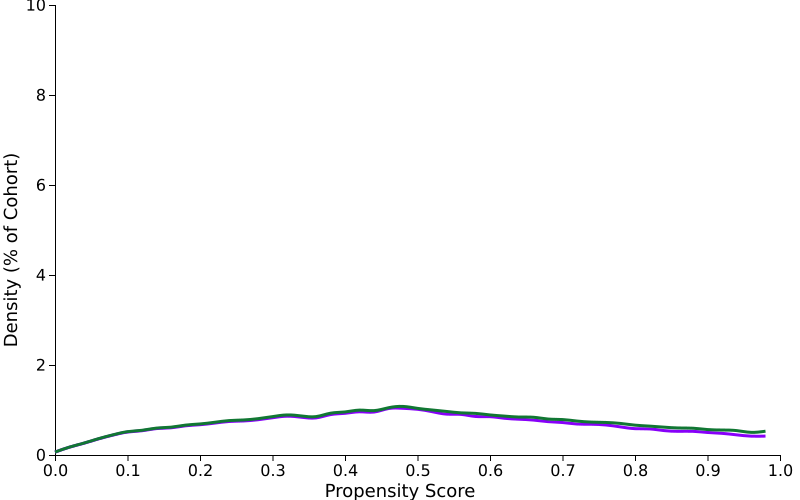 | |

Propensity score matching was performed on 28 characteristic(s). In the Demographics category patients were matched on Age at Index, Male, Not Hispanic or Latino, White, Black or African American, Hispanic or Latino, Female characteristic(s). In the Diagnosis category patients were matched on Atrial fibrillation and flutter, Heart failure, Other chronic obstructive pulmonary disease, Essential (primary) hypertension, Chronic kidney disease (CKD), Other peripheral vascular diseases, Atherosclerosis, Ischemic heart diseases, Diabetes mellitus, Cerebral infarction, Bicuspid aortic valve, Aortic aneurysm of unspecified site, without rupture, Personal history of nicotine dependence characteristic(s). In the Laboratory category patients were matched on Blood Pressure, Systolic, Cholesterol in LDL [Mass/volume] in Serum or Plasma, Hemoglobin A1c/Hemoglobin.total in Blood, BMI, Creatinine [Mass/volume] in Serum, Plasma or Blood, Left Ventricular Ejection Fraction (LVEF) (%), Activated partial thromboplastin time (aPTT) in Plasma or Blood, Prothrombin time (PT) in Plasma or Blood characteristic(s).

# **Supplementary Fig. 1**. Kaplan–Meier curves reporting freedom from aortic dissection according to Stanford classification.


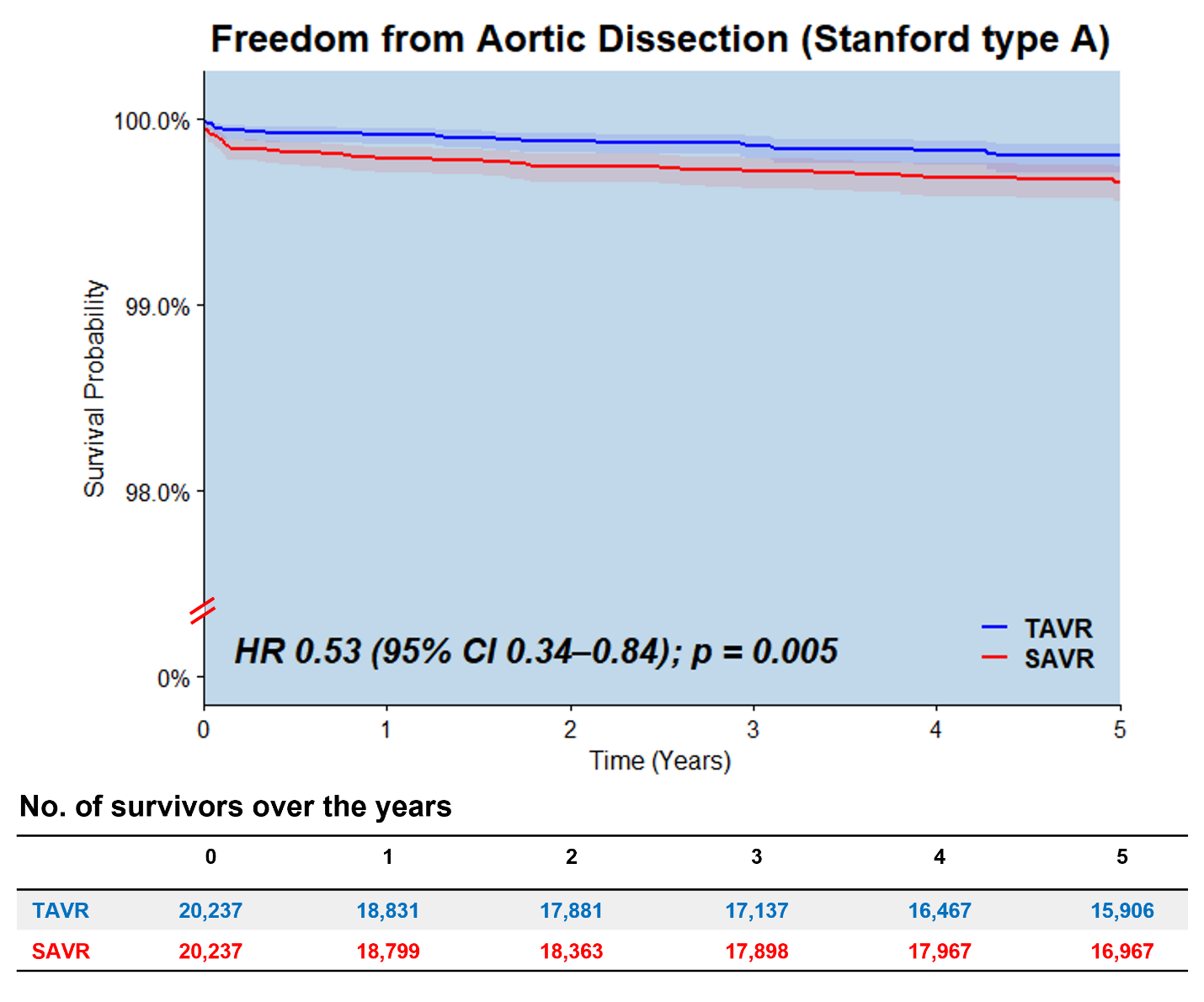


**Supplementary Fig. 1A.** Kaplan–Meier curves reporting freedom from aortic dissection (Stanford type A) across five years. HR: Hazard Ratio; TAVR: transcatheter aortic valve replacement; SAVR: surgical aortic valve replacement.


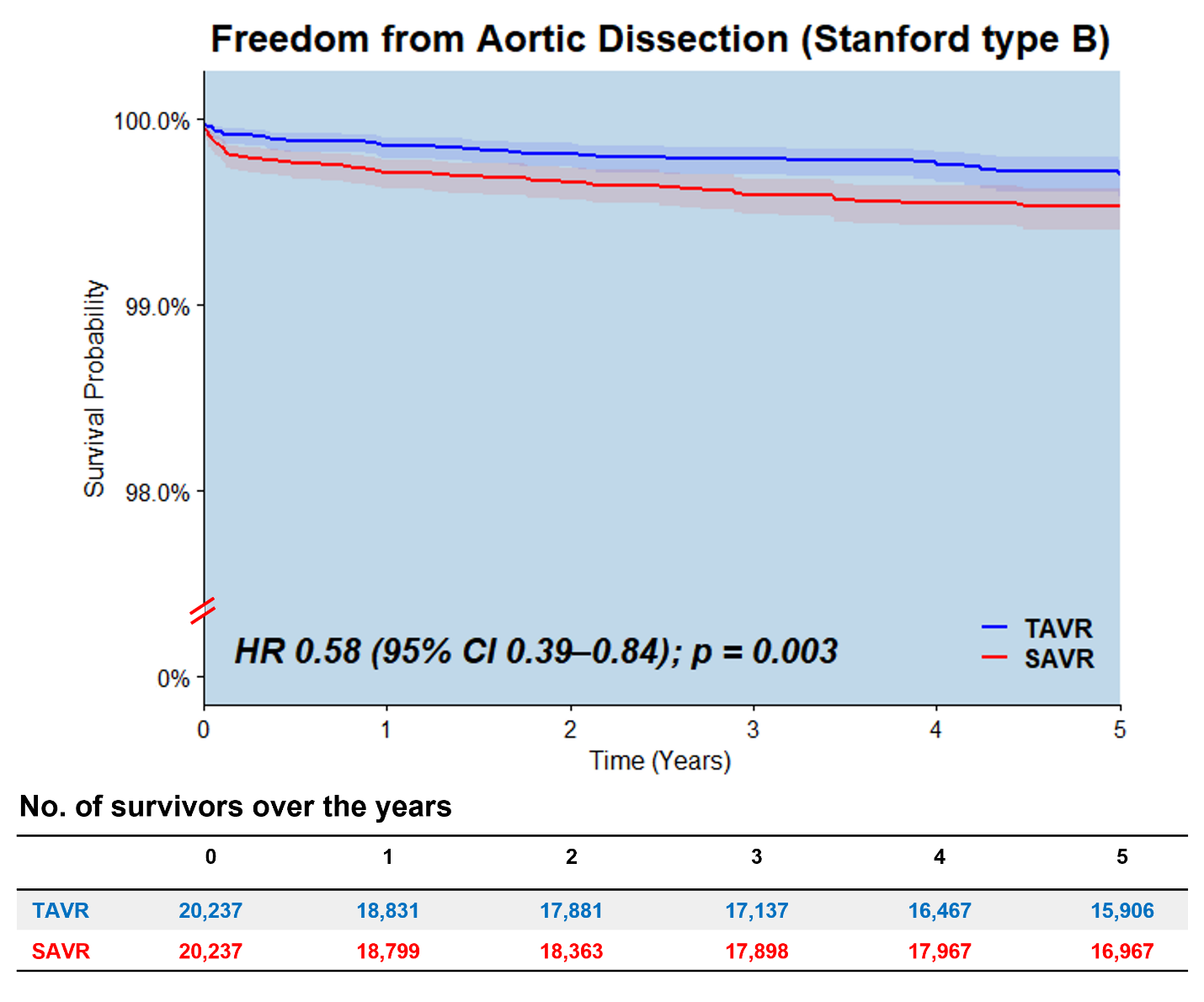
**Supplementary Fig. 1B.** Kaplan–Meier curves reporting freedom from aortic dissection (Stanford type B) across five years. HR: Hazard Ratio; TAVR: transcatheter aortic valve replacement; SAVR: surgical aortic valve replacement.
